# Effects of Parent Brand Equity on Perceived Fit and Customer Behavior of Extended Brand—Focused on MICE Destination

**DOI:** 10.3390/ijerph19084540

**Published:** 2022-04-09

**Authors:** Jiwon Lee, Eunjoo Yoon

**Affiliations:** 1 Program of Convergence Tourism Management, School of Future Convergence, Hallym University, Chuncheon 24252, Korea; jwlee04@hallym.ac.kr; 2 Department of Convergence Service Management, Hallym University of Graduate Studies, Seoul 06198, Korea

**Keywords:** brand extension, parent brand equity, perceived fit, place attachment, customer behavior intention, MICE destination

## Abstract

This study aims to examine the relationship among tourism destination brand equity (as parent brand), perceived fit, and customer behavior intention toward the extended MICE destination brand. It also identifies the moderating effect of place attachment between brand equity and customer behavior by adopting the brand extension concept in tourism and MICE destinations. The analysis of data collected from 381 respondents revealed that perceived fit is the most important factor influencing customer behavior, even though tourism brand equity and place attachment both had a positive effect on customer behavior, supporting all hypotheses. The theoretical implication of broadening the brand extension concept in MICE destinations and DMO marketing strategies is discussed.

## 1. Introduction

The Meetings, Incentives, Conventions, and Exhibitions (MICE) sector plays a significant role in the travel industry as a major driving force of the global economy. It is particularly important to many regions that have heavily invested in conventions, exhibition centers, and visitors’ bureaus [1]. Awareness of this economic potential has led numerous local and national governments to devote resources to the development or expansion of MICE facilities [2]. Moreover, in an increasingly competitive business environment, many cities have invested a large amount of money and labor in building bigger and better convention facilities. According to the Global Association of the Exhibition Industry (UFI), the global expansion of convention and exhibition centers has increased to a total of 1358 centers with a total space of 40.6 Mmsq as of 2022. Moreover, the Asian market with 41,160 sqm has the maximum average size of venue per region, led by China.

In this era of a highly competitive environment among MICE destinations, it is challenging for second movers who enter the market late to survive competition with top-tier MICE destinations. It seems futile to try to compete with those that have global brand power. Moreover, a brand extension strategy builds powerful brands and achieves important competitive advantages in the destination context. The brand extension includes strategies to maximize the value of a product (or service), and a destination brand is no exception [3]. It leverages the established existing brand name to launch new products and services instead of creating new brand names, reducing higher marketing risks and costs [4]. Thus, various studies in the marketing field have demonstrated that the strategy of brand extension enables the organization to expand its business into a new area in a cost-efficient way because of the security of a well-known parent brand [5]. However, an established body of research focusing on the brand extension concept within the tourism destination context is still lacking.

The success of brand extension depends on the fit between the parent and new brands, such as similarity and transferability between the two, while brand equity dimensions provide a useful metric to evaluate the impact of brand extension [2]. Thus, the evaluation of brand extension feasibility in a destination context should include perceived fit along with the strength of parent brand equity. When considering brand extension in destination branding, the most important aspect is that the new product (MICE) is an explicit part of the destination’s brand range, and there is consistency between concepts and features represented by the parent (tourism). MICE events serve as a link to tourism opportunities and present a powerful lever representing a tourism destination [6]. Moreover, the extant research also indicates that when customers have a favorable attitude toward a brand, they tend to positively evaluate the extension [4,7]. This is consistent with the research that determines the categorization of extended brands, such that when customers categorize an extension as a member of the parent brand category, their attitude and affect toward the latter is extended to the former and serves as the basis for the evaluation of the brand extension [8]. The rich set of schemas and affectively-laden memories linked to the target object of elevated attachment will be easily accessible to customers, thus increasing the salience of the parent brand association. Hence, attachment plays a prominent role in determining customer reaction to brand extension, but brand extension research is limited compared to the extensive studies in psychology.

Therefore, the goal of this study is to suggest a differentiated destination marketing strategy and competitive advantages for the second mover launching a new brand. This includes the MICE service, recognition as MICE destinations, and influencing the participants’ choices by adapting the brand extension concept. Based on the premise that established and newly emerged destinations can be conceptualized as “parent (herein, tourism)” and “extended (herein, MICE),” the relationship between parent brand equity, perceived fit, and customer behavior as an evaluation of extension will be examined. Moreover, the interaction effect between place attachment and brand equity on customer behavior is investigated, thus finding a distinctive role in brand extension evaluation. This study makes a conceptual contribution to the destination brand management literature by demonstrating the feasibility of a brand extension strategy in the tourism destination setting and discusses practical implications for destination management authorities and the MICE industry.

## 2. Literature Review and Hypotheses Development

### 2.1. Brand Extension in Tourism Destination

A destination brand is an overall impression of a destination on potential visitors including its functional and symbolic elements, encompassing the destination’s physical attributes, service, attractions, reputation, and benefits for visitors [6]. As the branding strategy intends to maximize the value of the product, the destination brand serves to maximize the value of the destination for visitors [9]. Several marketing studies in the destination or general product context are dedicated to investigating various kinds of branding strategies, one of which is a brand extension [3].

Brand extension includes the use of established brand names to launch new products. It is an increasingly popular leverage for new product introductions and is a prominent aspect of strategic brand development [10,11]. It refers to the stretching of the original (parent) brand into a new form that is considered more or less “true” to the original brand [12]. Specifically, by linking the new product to a known parent, parent brand knowledge is leveraged to establish a new product identity quickly and efficiently [13]. Hence, the extension is different but inherently connected to the parent brand as a result of shared product category, similar attribute target market, or usage situations [14]. Most figures, ranging up to 95% of all new products in the market, are some forms of extension [15]. Many companies employ product or service extension strategies for building and communicating strong brand positioning by enhancing awareness and quality associations [16]. Moreover, this increases the possibility of trials by decreasing new product risks while providing a new source of revenue [17]. Similarly, the pressure for destination management organizations to find cost-effective marketing strategies is increasing because of budget restrictions [18]. As the brand name on the extension is familiar to consumers, marketers typically spend less on the initial promotion of the extension than on introducing a new name. Therefore, brand extension can be employed in destination marketing as a viable and cost-efficient strategy. However, an established body of research focusing on brand extension within the specific context of destination marketing, or even in broader areas, ranging from commercial goods to service areas such as entertainment (film and game products), the hospitality (accommodation and food) industry, and customer perception, is still lacking.

For goods and services extensions, several factors have emerged from replicating and extending Aaker and Keller’s findings regarding the factors leading to brand extension success. These include the degree of fit between the parent brand and the extension category [10,19,20,21,22,23], consumers’ perceived quality of the parent brand [24,25,26], the number of other categories to which the parent brand has been extended [27], the consumers’ beliefs about the malleability of brand traits [28], the extent of consumer information, the nature of the competitive landscape [29], and the depth of association of the parent brand with a given category [30]. Likewise, the brand extension concept could be feasible in destination contexts. Tourism literature describes destinations as places or regions in spatial hierarchy that relay images and narratives described in terms of the destination’s attraction, facilities, and service [31]. MICE events and tourism are distinct but closely tied services provided by the destination to visitors who want to experience a certain different service during their stay. Even though destination authorities have occasionally used a MICE event as a gadget to attract more visitors, it would be successful under the premise that enjoyable attractions exist after the event [32]. That is, MICE visitors will have a more favorable image of the places having a high reputation and image regarding tourism resources. As tourism and MICE resources are complementary, sharing a similar resource destination, a visitor may recognize perceived fit or similarities leading to the brand extension effect.

Despite this, there are few studies related to brand extension in the hospitality context. Prados-Peña and del Barrio-García studied the extension of the heritage site into the hotel brand (high fit) and clothing brand (low fit) to examine the customer’s attitude toward the extension of a cultural heritage brand. They concluded that the impact of fit on consumer attitudes depended on the complexity of extension [33]. Kim et al., analyzed that brand parent equity and perceived fit influenced extended brand equity and demonstrated the feasibility of “destination extension” using an experimental design with two South Korea and UNESCO World Heritage sites as parent brands and Jeju Island as the extended brand [3]. According to Chalip and Costa [6], an event marketed under its host destination’s brand will be received more favorably if it is conceptually consistent with other elements in the destination product and service mix. They also insisted that the fundamental advantage of an event that can be treated as a brand extension is that it is closely tied to the host destination’s brand. Furthermore, the destination market share would increase with the tourist’s enhanced perception of the brand image and value when the event is favorably received by the market. Accordingly, it is explicable to regard tourism services as parent brands and MICE services as extended brands in the destination marketing context by applying the firm’s product extension strategy to the destination brand.

### 2.2. Relationship among Parent Brand Equity Perceived Fit and Extended Brand Behavior Intention

Keller defined brand equity as the impact of marketing on a customer’s knowledge of a brand and their reactions to that knowledge [34]. Using and adopting brand equity to understand consumer behavior, Yoo and Donthu [35] suggested that it is a combination of cognitive dimensions (brand association, brand awareness, and perceived quality). Considering the significant influence of brand equity on consumer preferences and attitude, the brand equity construct was employed to measure the relationship between consumer loyalty, satisfaction, and purchase intention [36]. Realizing the growing importance of brand equity in promoting the destination, later studies received relatively minimal attention related to destination brand equity in the tourism field [37,38] and incorporated findings based on product brand equity into the field [35]. These studies show that, in many brand extensions, brand equity has been discussed as an important factor [27,39]. However, research on the transfer of brand equity in the process of developing a new destination brand using the parent brand is still lacking. Thus, this study attempts to explore whether the brand equity of the parent brand can be transferred to an extended service brand in the context of destination brand extension.

Several factors determine the success of brand extension. The most important factor identified in prior research is perceived fit [40]. Fit is defined as the extent to which the image, associations linked to the parent brand, and the extension product are similar and integrate well [41]. Based on the theories of categorization, and belief or affect transfer, the perceived fit between the two brands has been the focus of several studies examining the customer evaluation of brand extension [42] and the extension’s effect on the parent brand [43,44]. Generally, the better the perceived fit, the better the consumers’ evaluation of the extension because of the credibility of the new product [4,7].

Theoretically, a direct relationship between brand extension attitude and change in brand equity is embedded in schema-change mechanisms that are assumed to occur within a consumer’s existing knowledge base (schema) of a parent brand [45]. Brand knowledge or schema is conceptualized as an associative network of memory nodes, where each node represents a brand-related concept and the linkages among the nodes represent brand associations [34]. Whenever a brand extension is launched, it creates new memory nodes and a link back to a parent brand [46]. For example, when consumers encounter a new brand extension, they are likely to evaluate it based on their existing knowledge of the product category as well as the attributes (e.g., familiar image) of the parent brand, thus reducing the perceived risk for the new product [47]. Reduced risk enhances the customer expectation and inference of the same quality toward an extended brand with the parent brand, and, in turn, acquires the suitability for extension [48]. Dillon et al., reported that consumer perception of brand image impacts purchase and consumer evaluation of a brand’s new products [49]. Salinas and Pérez examined the positive effect of parent brand image on brand extension [50]. Völckner and Sattler mentioned that the extent to which consumers find a parent brand to be “likeable” and “relatable” can impact the extent to which new brand extensions are positively evaluated [10] p. 32. Moreover, interpersonal or extrinsic benefits (e.g., perceived brand quality) of brand consumption are pivotal when consumers communicate with each other about their brand experience, and research shows that brand extensions can inherit such benefits [51]. Trzonkowski attempted to prove, through interviews, that sport events were regarded as extended destination brands and outdoor activity was considered as a parent destination brand [32], explaining the possibility of adopting the brand extension concept to the MICE event area. Favorable brand association with the overall parent brand could improve brand equity, thus enabling tourism destination to increase the number of visitors including MICE event visitors via positive emotional transferring formed by a tourism experience to the MICE event.

Accordingly, in the presence of positive attributes, consumers develop positive brand attribute associations when transferred to a brand extension and enhance the evaluation by inferring the quality or benefit of a new brand [52]. Thus, we expect in the tourism destination context that visitors who have a positive and strong recognition of the existing tourism destination brand equity will favor new brands such as MICE events and intend to visit them. This expectation leads to:

**Hypothesis** **1** (**H1**)**.** *Tourism destination equity has a positive impact on the perceived fit between tourism as a parent brand and MICE as an extended brand in the destination context.*

**Hypothesis** **2** (**H2**)**.** *Tourism destination equity has a positive impact on the behavioral intention of MICE as extended brands in the destination context.*

While the brand equity dimension provides a useful metric to evaluate the impact of brand extension on the product, its success depends on the similarity or fit between the parent brand and the new product [53]. Consistent with categorization theory, prior brand extension studies posit that the degree to which consumers transfer their parent brand associations to an extension depends on the level of perceived fit or similarity between the extension category and the parent brand. Specifically, consumers evaluate extensions more favorably if the perceived fit between the parent brand and extension is high. Thus, it is evident that the most frequently considered antecedent of the brand, as several studies [7,27,43] have reported, is that the greater the fit between the original and extended brands, the greater the transfer of positive affect. Visitors recognize the similarity between a MICE event and tourism, since a MICE event shares tourism attributes as a hedonic value affecting visitor satisfaction. Additionally, visiting intention and its similarity encourages a visitor to have favorable association transferring to the new service: MICE from tourism experience. Based on the above reasoning, the hypothesis is as follows:

**Hypothesis** **3** (**H3**)**.** *The perceived fit between tourism as a parent and MICE as an extended brand has a positive effect on the behavioral intention of extended MICE brands in the destination context.*

### 2.3. Place Attachment in Brand Extension

The visitors who feel highly attached to the place are highly invested in the location, are willing to patronize the establishment, and intend to tell others about the place [54]. As attachment is an affective and emotional feeling, the person–place bond advanced the foundations of place attachment theory [55]. Initially conceptualized as “an affective bond”, place attachment has developed into a complex concept involving “the interplay of affect and emotions, knowledge and beliefs, and behaviors and actions” [56], p. 155.

Tourism literature provides evidence that place attachment significantly contributes to the understanding of tourist behavior [56,57]. Although scholars of tourism have analyzed place attachment as a multidimensional construct [58], many studies have focused on the interrelationship between only two dimensions of place dependence and identification [59,60,61,62]. Place dependence refers to the extent to which a consumer depends on the establishment to meet their needs, physical, social, or emotional, in the form of connection, friendship, and a sense of belonging. Place identity measures the extent to which consumers perceive that their identity is in some way tied to that of the business, or that the patronage of the establishment makes up a large part of their self-concept [63].

Affect as attitude is transferred from the parent brand to its extension and serves as a base for the brand extension evaluation; consequently, positive attitudes are transferred to the extension if an attachment exists in the parent brand. Stronger attachment to a particular target (i.e., object, place, or person) induces a state of emotion-laden mental readiness that affects the allocation of emotional, cognitive, and behavioral resources toward the object [64]. A highly relevant connection associated with attachment results in the formation of a set of schemas, exemplars, and affectively-laden memories linked to the object [65] due to the enhanced motivation to devote cognitive, emotional, and behavioral resources to the target attachment [66]. These will be easily accessible to consumers, thus increasing the salience of the parent brand associations [8]. As a result, we can expect the attachment to make consumers more impulsive toward extensions and to play a prominent role in determining consumer reactions to brand extension. Extant research posits that in evaluating extension, consumers first go through the categorization stage and via evaluation or affect, they move to the transfer state. Moreover, as customers with high brand attachment are proactively involved in corporate marketing activities and wish to have long-term brand relationships, brand attachment is a prominent variable for brand extension strategy [67]. Despite the significant role of attachment in brand extension, research on this topic is scarce.

Lee and Chang found that customers with a higher brand attachment and a more intimate self-relationship showed a more favorable attitude toward the extended brand, proving the moderating effect of brand attachment [68]. Lee and Moon investigated a customer with a high degree of brand attachment who positively evaluated the extended product, irrespective of the degree of fit to the parent brand [69]. Fedorikhin et al., revealed that customers with elevated levels of attachment to the parent brand are willing to pay more for brand extension, forgive the brand mishaps, and recommend it to others even when the fit is moderate, by supporting the potential strength and importance of attachment constructs [8]. Furthermore, Thorbjørnsen estimated that a customer with a higher familiarity with the brand has a negative attitude toward the inconsistent concept of extension [70]. Moreover, customers with higher familiarity show a more positive attitude towards the extension than customers with lower familiarity. Thomson et al., stated in their research assessing consumer’s emotional attachment to brands that strong emotions invoked for the attached object (parent brand) reflect one’s evaluation reactions to the extended object without any direct contact or experience with it [67]. Based on the above reasoning, the hypothesis is as follows:

**Hypothesis** **4** (**H4**)**.** *The interaction between tourism brand equity and place attachment has a positive impact on the behavioral intention of MICE as extended brands in the destination context.*

## 3. Methodology

### 3.1. Research Design

**Figure 1:**
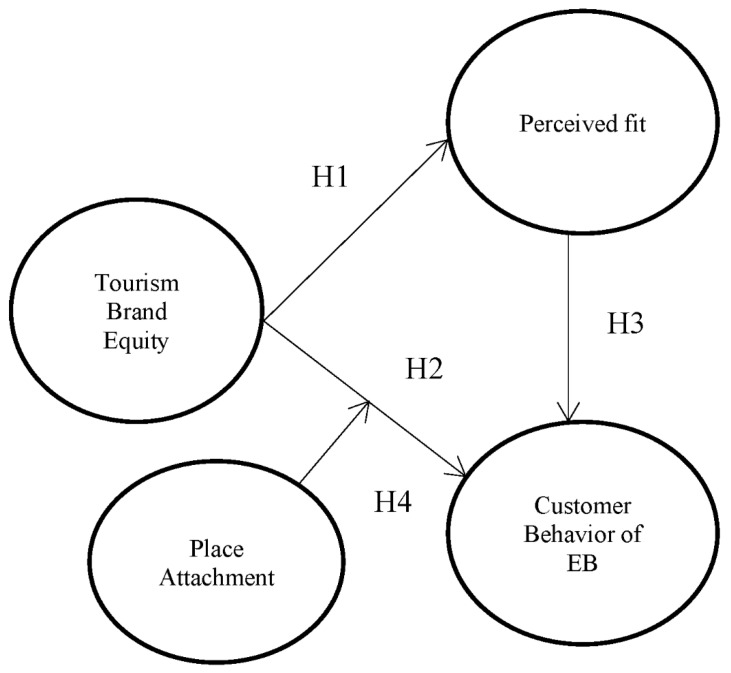
Research model.

This study was designed to evaluate the relationship between parent brand equity, perceived fit, and behavior intention of extended brands focused on the destination brand (refer to Figure 1). Those who visited Gangwon Province in Korea for tourism or business and experienced at least one MICE event participated in the survey. Gangwon Province is one of the top-tier tourism destinations of Korea but is a second mover as a MICE destination, following Seoul, Jeju, and Busan, the top-tier MICE cities in Korea. Gangwon Province is an optimum sample to find a marketing strategy for brands competing with top-tier MICE cities using parent brand equity’s extension effect on the new brand of the MICE city. The researcher collected the visitors’ email and contact information from the MICE event calendar data and event hosts and surveyed approximately 500 people who agreed to participate in the study from 24 December 2016 to 5 February 2017, using Google forms. A total of 381 usable responses were collected, yielding a response rate of 76%, and analyzed using structural equation modelling (SEM) to investigate the causal effects among three factors (parent destination brand equity, perceived fit, and behavior intention of the extended brand) and the moderating effect of place attachment between parent brand equity and customer behavior of extended brands.

### 3.2. Measurement Items

The study used established measurements from previous research tailored to fit the context of destination brands. The commonly accepted and widely used scale was used to measure the constructs of interest in the study. Parent brand equity estimated three constructs, brand image, awareness, and perceived quality, with 11 items defined as intangible value, which has a dominant position other than the competitive destination and the differentiated effect of tourism destination recognized by visitors and developed by Yoo et al. and Nam et al. [71,72]. Regarding place attachment, identity, and dependence, each of the four items was assessed using items adapted from Kyle and Mowen and Yuksel et al. [56,73]. The perceived fit scale was measured using three items adapted from Aaker and Keller’s scale [4] by defining the degree of similarity between parent and extended brands. Behavior intention was measured using three items adapted from the scale developed by Zeithaml et al. [74], consisting of visiting intentions, possibilities, and recommendations. All items included in the survey instrument were measured on a 5-point Likert scale, ranging from 1 (“strongly disagree”) to 5 (“strongly agree”). Finally, demographic variables (e.g., gender, age, education, and occupation) were measured.

## 4. Results

### 4.1. Sample Profile

Approximately half (47%) of the 381 respondents were male and 43.8% were in their 30s followed by those in their 40s (24.9%) and 20s (16.8%). Regarding education, 66% of the respondents were university graduates and 22% had a bachelor’s degree or above. Regarding occupation, 39.6% of respondents had an office job, and 12.6% were professionals. The majority of the respondents (25.7%) had work experience between five to ten years and 22.3% had worked for more than ten years.

### 4.2. Factor Analysis and Reliability Tests

IBM SPSS Statistics for Windows, version 23.0, was used for the following tests. Initially, an exploratory factor analysis (EFA) harnessing principal component analysis and the varimax rotation method were employed to determine the a priori dimensionality of each construct. The Kaiser–Meyer–Olkin (KMO) test and Bartlett’s test of sphericity were computed to assess the appropriateness of the factor analysis of the data. The KMO measure of sampling adequacy was 0.922, 0.733, 0.739, and 0.922 for parent destination brand equity, perceived fit, customer behavior of extended brand, and place attachment, respectively. Moreover, Bartlett’s test of sphericity for the above constructs was significant (*p* < 0.001), verifying the adequacy of EFA use [75]. To assess the reliability and validity of the reflective measures, the outer loading values needed to be above 0.4, based on Hair et al. [76]. The reliability of the measure was demonstrated by Cronbach’s alpha and composite reliability values exceeding 0.7. The constructs had good internal consistency (see Table 1).

### 4.3. Confirmatory Factor Analysis (CFA)

A confirmatory factor analysis (CFA) was conducted to assess the overall model fit of the measurement model and specify the relationships between the observed variables and four latent constructs using maximum likelihood estimation. Assessment of a variety of goodness-of-fit measures to evaluate the overall model fit showed the following results: χ^2^ (36) = 77.453 (*p* < 0.000, df = 36), GFI = 0.9612, NFI = 0.974, CFI = 0.986, RMR = 0.016, RMSEA = 0.055. All goodness-of-fit indices were within the acceptable limits [75]. The composite construct reliability (CCR) values were greater than the threshold of 0.70, as proposed by Fornell and Larcker [77]. Convergent validity was assessed using the average variance extracted (AVE) of all constructs in the measurement model and demonstrated with a value above 0.5 [77]. Moreover, the AVE for each construct was greater than the squared correlation coefficients for the corresponding inter-constructs, and the measurement model was deemed acceptable regarding construct reliability and discriminant validity (see Table 2 and Table 3).

### 4.4. Result of the Structural Model

The structural model was used to empirically test Hypotheses 1 to 3, and the results are shown in Table 4. According to the fit indices based on AMOS, the model provided an acceptable fit for the data (χ^2^ (24) = 77.156, *p* < 0.000, GFI = 0.901, CFI = 0.949, NFI = 0.936, RMR = 0.034, RMSEA = 0.097), and the results reveal that the overall fit of the structural model was satisfactory [78]. For Hypothesis 1, tourism destination brand equity was positively associated with the perceived fit of extended MICE destination (β = 0.736, *t* = 11.729, *p* < 0.001). Tourism destination brand equity also showed a positive relationship with customer behavior for extended MICE destination brand (β = 0.153, *t* = 2.388, *p* < 0.05), supporting Hypothesis 2. The tourism destination (parent brand) brand equity had an indirect effect on customer behavior mediated by the perceived fit of the extended brand (β = 0.112, *p* < 0.05). Finally, the perceived fit of the extended MICE destination brand was positively associated with customer behavior (β = 0.781, *t* = 10.405, *p* < 0.001), supporting Hypothesis 3.

### 4.5. Moderating Effect Analysis

This study mainly aimed to examine the interaction effect (moderation) of place attachment on the interrelationships between tourism destination (parent brand) brand equity and customer behavior of extended destination brands. The stronger the dependence and identification of the place, asking to extend the stay, and telling others about the good experience of the place, the more favorable the attitude toward any objects within the destination by inducing emotion-laden mental readiness. If the place affect is added to anyone who had existing high destination equity, their favorable attitude for extended service and MICE will be more maximized [68]. To estimate the influence of place attachment with the above variables, SEM was employed according to the findings of Chin et al. [79]. Product indicators, in which interaction terms were computed by multiplying the predictive and moderating variables, were used. Before introducing the interaction effect, it was verified that path coefficients represent the relationship between tourism destination brand equity and customer behavior moderated by place attachment. The results shown in Table 5 indicate that all hypothesized relations significantly affect customer behavior, supporting Hypothesis 4.

## 5. Discussion

It is important to learn how to survive in a highly competitive situation where many MICE destinations emerge regularly and desperately compete to attract business events. As the competition among destinations is increasingly fierce, it is necessary to identify factors that contribute to competitive advantages [80] and effective differentiation from destinations with similar characteristics that are easily substituted [35]. The brand extension strategy has been shown to build powerful brands and achieve essential competitive advantages [5]. This study intended to investigate the structural relationships among tourism destination brand equity (as parent brand), perceived fit, and customer behavior of an extended MICE destination brand, as well as the moderating role of place attachment in the brand extension theoretical framework, supporting all hypotheses. This study provides important results and contributes to the understanding of brand extension strategies in the tourism destination context.

First, it confirmed that tourism destination brand equity, including brand image, awareness, and perceived quality, was an important element in stimulating the perceived fit (β = 0.736), which increases the positive value and reduces the negative outcomes of the new brand [81]. Moreover, it showed the positive effect of customer behavior directly and indirectly (β = 0.153 and 0.122, respectively) through the perceived fit even for the extended destination brand with the assumption that a new extended service would have the same equity. This confirms that when customer’s parent brand evaluation is more favorable, extensions are more likely to be successful, which is in line with the findings of prior studies [4,13,23,53] thus realizing the importance of parent brand management.

Second, we found that perceived fit (β = 0.780) is a more important factor than parent brand equity (β = 0.153) in brand extension regarding customer behavior towards extended brands.

This supported and clarified past research on the importance of fit for new brand extensions. It explained that the customer’s perception of substitutable, complementary, and transferable attributes between the current service and the new service brand offered by the destination plays a key role in their destination marketing strategy when launching a new service.

Finally, this study showed that emotional bond is a crucial element for destination brand extension. It was estimated through the interaction effect (β = 0.122) between place attachment and parent brand equity on the customer behavior towards extended brands. Some studies found that higher product attachment could make customers invest more time; they also try to transform the product for individualization, not showing reluctance for new products/services within their brand concept territory [82]. As a result, a visitor who has a strong bond with the place has a favorable attitude, revisiting the newly branded destination since it keeps the customers’ relationship strong with the brand. Although the direct effect of tourism destination brand equity is much stronger (β = 0.532) than place attachment (β = 0.269) on customer behavior, this study supports the existence of the effect of place attachment on customer behavior through moderating effect analysis.

## 6. Conclusions

Brand extension research has been pervasive in products and service areas, including food and hotel service industries, but it has not been given attention in the tourism destination context. Few conceptual research [83] and empirical studies [3,84] have applied brand extension theory to the destination context. From a theoretical perspective, first, this study widened the brand extension concept in destination management research. This was carried out empirically by examining the relationship between tourism destination brand equity and customer behavior of extended destination brands in MICE services. Traditionally, marketing strategies for MICE destination management were mainly to emphasize the choice attributes for MICE venues, facilities, and infrastructure. However, it is not easy for the second mover to compete equally with the top-tier MICE venues such as Seoul, Jeju, and Busan, which have high brand power (equity) as global MICE destinations. Second, this research theoretically proved that emphasizing tourism resources, attractive places, and unique social and cultural heritage is useful for enhancing the MICE brand attitude. Specifically, Gangwon Province, Korea, which is a top-tier tourism destination with various tourist attractions such as beautiful natural resources and treasures, leisure spots, historical destination, and open space for culture, drama and films, seems to be the optimum research target to verify brand extension in the destination context. Third, this empirical investigation of the interaction effect of place attachment in destination brand extension contributes to broadening the theoretical framework in the destination management literature. The extant research indicates that when customers have a favorable brand attitude, they tend to positively evaluate extensions [4,7]. Nevertheless, customer attachment and emotional-laden relationships between customers and brands have rarely been introduced in the brand extension literature. Moreover, a lack of studies is observed regarding the effect of place attachment on parent brand equity and customer behavior of extended brands in the tourism destination context. Lastly, this study contributes theoretical support for the “brand architecture” concept adopted by the “place brand portfolio” developed by Dooley and Bowie [38]. It states that the trust and value of the country brand as an umbrella brand acts as a guarantee for subordinate brands such as region. It also influences customer attitude or association by sharing or delivering the values—reliability, reputation, and quality [85]. When tourism is regarded as an umbrella brand of the region, MICE events or other activities could be regarded as extended subordinating brands influenced by the fame of the region. The results offer several important managerial implications. First, it confirmed the pivotal role of parent brand equity, tourism destination image, awareness, and quality, and its impact on the perception of fit for extension and customer behavior of new brands. Accordingly, destination marketers should recognize the need for policies to establish their differentiated brand equity, to “be like” the place (here in Gangwon), primarily as an original brand and to make the brand image a familiar city. For example, focusing on attracting business events associated with brand image, such as cultural, environmental, and natural heritage, is more strategic rather, than having reckless competition with other top-tier MICE cities. As a result, Gangwon Province has succeeded in attracting events such as the World Forest Expo 2022, Art Festival, and ITS World Assembly, 2026.

Second, since this study proved that the perceived fit of substitutable, complementary, and transferable service attributes between the tourism and the MICE event is the most important factor in destination extension, the authorities need to emphasize the fit between the MICE and the tourism for the MICE destination brand strategy. It also supports that their decision to announce the MICE venue since 2015, along with the establishment of Gangwon CVB, is reasonable by creating additional value for the destination by extending to the MICE service brand associated with the tourism destination.

Finally, this study showed that brand attachment can increase brand extendibility. It is an important explanatory variable for successful extensions, and hence, destination marketers need to explore ways to make visitors spend more time at the destination, increase their satisfaction during the stay, and make the tourism experience favorable, thereby achieving the successful destination brand extension as a result. For example, local planners, public services, and individuals can work together to develop tourism destinations that better reflect visitors’ desires to increase their emotional attachment through social media activity, such as inviting and encouraging the visitor to post comments on social networking site platforms, thus facilitating the delivery of stories about the overall tourism experience.

As with any empirical study, this study has some limitations, which can be turned into opportunities for further research. First, this study did not fully examine the interrelationship among every factor in this model due to the lack of existing literature with empirical verification. Thus, it leads to less innovation or a lack of novelty. However, broadening the theoretical framework to a new industry is meaningful as initial work and provides the motive for further investigation. Moreover, the individual effects of brand equity, brand image, awareness, and quality on the brand extension were not scrutinized, thus narrowing the research comprehension. Future studies should aim to develop multiple variables to measure the role of parent brands in destination extensions. Second, this study surveyed a specific area, Gangwon Province, Korea, which may limit the ability to generalize these results to other destination extensions, even though it is perfect for applying the brand extension concept in the MICE destination. Thus, further empirical research including various destinations and outcome variables would be required for generalization and more objective comprehension.

## Figures and Tables

**Table 1:** Result of exploratory factor analysis (EFA).

Construct	Factors	Measures	Factor Loading	Variance (%) (Cronbach’s α)
Parent	Perceived Quality	Consistent service providing	0.815	53.238 (0.855)
		High service quality	0.810	
		Excellent tourism infrastructure providing	0.732	
		Reliable as tourism destination	0.729	
Brand	Brand Image	Differentiated personality	0.835	10.082 (0.856)
		Associated unique image	0.772	
		Familiar image as tourism destination	0.612	
		Reliable image	0.605	
		Associated as something refreshing	0.547	
Equity	Awareness	Popular as tourism destination	0.851	6.732 (0.726)
		Easily occurred as tourism destination	0.791	
		Easily distinguishable from other cities	0.709	
KMO = 0.922, Bartlett’s test = 2557.084 (*p* < 0.001), Total variance, 70.051%				
Place	Identity	This place means a lot to me	0.815	59.286 (0.859)
		I am very attached to this place	0.798	
		Place tells who I am	0.760	
		Able to tell many things to others	0.743	
Attachment	Dependence	Feel special things that cannot be felt in another place	0.817	10.181 (0.836)
		Impossible to be replaced by another place	0.771	
		Optimum place as tourism venue	0.704	
		Satisfied with the visit to place	0.696	
KMO = 0.902, Bartlett’s test = 1608.934 (*p* < 0.001), Total variance 69.467%				
Perceived Fit		MICE event held represented the city brand as tourism destination	0.796	77.697 (0.856)
		Tourism is complementary to MICE	0.774	
		Tourism can be used as resource for MICE	0.761	
KMO = 0.733, Bartlett’s test = 510.560 (*p* < 0.001), Total variance 77.697%				
Customer Behavior Intention		Visiting intention	0.911	80.693 (0.880)
		Able to visit the place	0.902	
		Recommendation	0.882	
KMO = 0.739, Bartlett’s test = 610.563 (*p* < 0.001), Total variance 80.693%				

**Table 2:** Result of Confirmatory Factor Analysis.

Constructs	Measures	Std. β	*t*-Value	C.R	AVE
Tourism Destination Brand Equity	Perceived quality	0.761	-	0.92	0.79
	Image	0.866	17.220 ***		
	Awareness	0.759	15.748 ***		
Place Attachment	Identity	0.772	-	0.90	0.81
	Dependence	0.916	17.459 ***		
Perceived Fit of Extended Brand	Substitute	0.823	17.590 ***	0.88	0.71
	Complementary	0.825	17.648 ***		
	Transferability	0.800	-		
Customer Behavior Intention	Intent to visit	0.855	19.647 ***	0.91	0.78
	Able to visit	0.848	19.426 ***		
	Intent to recommendation	0.827	-		
Model fit: ꭓ^2^ (35) = 77.453 (*p* < 0.000). GFI = 0.962, NFI = 0.974, CFI = 0.986, RMR = 0.016, RMSEA = 0.055					

*** *p* < 0.001.

**Table 3:** Discriminant validity.

Constructs	TDBE	PA	PF	CBI	AVE
TDBE	1				0.79
PA	0.77	1			0.81
PF	0.57	0.52	1		0.71
CBI	0.54	0.51	0.70	1	0.78

Note: TDBE: tourism destination of brand equity, PA: place attachment, PF: perceived fit, CBI: customer behavior intention.

**Table 4:** Result of SEM.

Hypothesis	Path	Path Estimate	*t*-Value	Status
H1	TDBE → PF	0.736	11.729 ***	Supported
H2	TDBE → CBI	0.153	2.388 **	Supported
H3	PF → CBI	0.781	10.105 ***	Supported
Indirect Effect	TDBE → PF → CBI	0.122	*p*-value: 0.009	
Model Fit: ꭓ^2^ (24) = 77.156 (*p* < 0.000), GFI = 0.954, CFI = 0.976, NFI = 0.966, RMR = 0.02, RMSEA = 0.076				

** *p* < 0.05, *** *p* < 0.001; Note: TDBE: tourism destination of brand equity, PA: place attachment, PF: perceived fit, CBI: customer behavior intention.

**Table 5:** Result of moderating effect.

Hypothesis	Path	Path Estimate	*t*-Value	Status
H4	TDBE → CBI	0.532	3.130 **	Supported
	PA → CBI	0.269	1.762 *	Supported
	TBDE x PA → CBI	0.0122	2.450 **	Supported

* *p* < 0.01, ** *p* < 0.05, Note: TDBE: tourism destination of brand equity, PA: place attachment, CBI: customer behavior intention.

## Data Availability

Not applicable.
